# Viral manipulation of the HLA class I antigen processing and presentation pathway

**DOI:** 10.3389/fimmu.2025.1694498

**Published:** 2025-12-05

**Authors:** Dominic S Nolan, Arwen F Altenburg

**Affiliations:** Department of Pathology, University of Cambridge, Cambridge, United Kingdom

**Keywords:** HLA, immune evasion, virus, infection, antigen presentation, CD8+ T cells, NK cells, MHC

## Abstract

Major histocompatibility complex (MHC, human leukocyte antigen [HLA] in humans) class I molecules present peptides at the cell surface to cytotoxic immune cells. Peptides are generated and selected for MHC class I presentation in the antigen processing and presentation (APP) pathway. Recognition of a foreign peptide in the context of MHC class I by CD8^+^ T cells results in target cell lysis. While CD8^+^ T cells don’t provide sterile immunity, they are pivotal in viral infections by clearing infected cells and may impact disease duration and severity, and virus spread. Additionally, MHC class I molecules act as ligands for NK cell receptors, which similarly play an important role in the control of virus infections through cytotoxic activity and cytokine production. To evade immune recognition, viruses have developed strategies to modulate MHC class I levels by targeting MHC molecules directly or by disrupting components of the APP pathway. Herpesviruses, large DNA viruses that encode numerous immunoevasins, are notorious for disrupting virtually every stage of the MHC class I APP pathway. Over the years, it has become clear that a wide range of other viruses also have evolved targeted mechanisms to modulate MHC class I or components of the APP pathway to evade cytotoxic immune responses. Here, we review the literature on targeted viral manipulation of HLA class I, including non-classical HLA molecules, and modulation of components of the APP pathway by viruses infecting humans.

## 1 Introduction

### 1.1 The MHC class I antigen processing and presentation pathway

Presentation of immunogenic peptides on major histocompatibility complex (MHC, human leukocyte antigen [HLA] in humans) class I is crucial for immune detection and control of malignant or infected cells. MHC class I molecules consist of a heavy chain (HC) bound to β_2_-microglobulin (β_2_m). Assembly of these molecules requires several chaperones that aid in general protein folding and quality control in the endoplasmic reticulum (ER). The immunoglobulin-binding protein BiP (aliases HSPA5 or GRP78) stabilises polypeptides during folding, facilitates retrograde translocation of misfolded proteins for proteasomal degradation, and regulates the unfolded protein response (1, 2) (**Figure 1.1**). The chaperone calnexin recognises monoglycosylated N-linked oligosaccharides on folding intermediates, assists in folding, and releases the MHC class I HC when it assembles with β_2_m (1, 3–5) (**Figure 1.2**). ERp57 (aliases PDIA3 or GRP58) is recruited by calnexin and catalyses disulphide formation in the HC (5–7).

**Figure 1:**
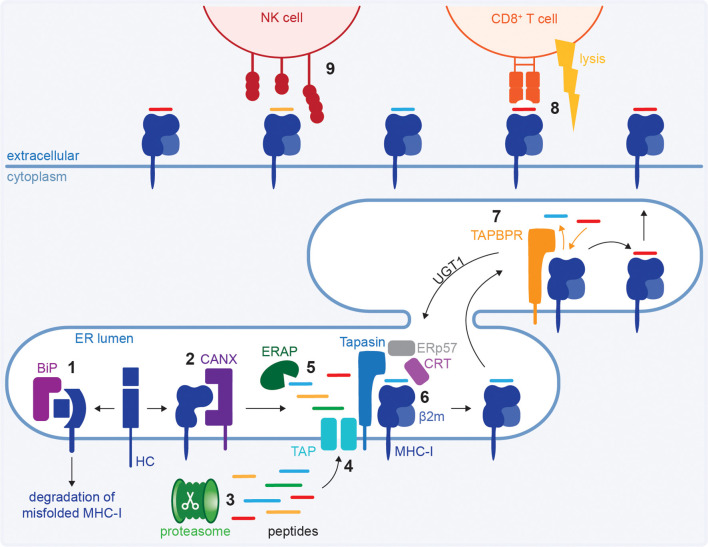
The MHC class I antigen processing and presentation pathway. MHC class I heavy chains (HC) are translated into the ER where BiP performs quality control **(1)** and folding is assisted by calnexin (CANX) and ERp57 **(2)**. Proteins are degraded by the proteasome **(3)** and peptides are transported into the ER by transporter associated with antigen processing (TAP) **(4)** where they can be further trimmed by ER aminopeptidases (ERAP) **(5)**. Tapasin loads a peptide onto MHC class I in the peptide loading complex (PLC) **(6)** and TAPBPR further optimises the peptide repertoire **(7)**. Optimally loaded MHC class I molecules traffic to the cell surface for immune surveillance. When a CD8^+^ T cell recognises a foreign peptide in the context of MHC class I, it lyses the target cell **(8)**. Additionally, MHC class I molecules serve as a ligand for NK cell receptors **(9)**.

Immunogenic peptides for presentation on MHC class I are generated by proteasomal processing (**Figure 1.3**) of proteins that are degraded because of misfolding, as part of their natural turnover (‘retiree’), or are sourced from defective ribosomal products (DRiPs), which are defective polypeptides produced from errors occurring during translation (8, 9). Peptides are transported from the cytoplasm into the ER by the transporter associated with antigen processing (TAP) (10–13) (**Figure 1.4**), where they can be further trimmed to an optimal length of 9–12 amino acids by ER aminopeptidases (ERAPs) (14–17) (**Figure 1.5**).

To acquire peptides, MHC class I molecules are chaperoned by calreticulin, a soluble homologue of calnexin (18). Calreticulin recruits the HC:β_2_m heterodimer into the peptide loading complex (PLC), which additionally consists of tapasin docked onto TAP, and is stabilized by ERp57 (19–23) (**Figure 1.6**). Peptides are loaded onto MHC class I by tapasin in the PLC (24–28). MHC class I molecules can undergo further peptide editing by TAPBPR, a tapasin homologue that functions independent of the PLC (29–31). TAPBPR can perform direct peptide exchange or recruit UDP-glucose:glycoprotein glucosyltransferase 1 (UGT1), which causes peptide receptive MHC class I to recycle back to the PLC for peptide acquisition (32, 33) (**Figure 1.7**).

Stable peptide:MHC class I complexes traffic to the cell surface for immunosurveillance by cytotoxic immune cells. A foreign or modified peptide in the context of MHC class I activate cytotoxic CD8^+^ T cells which subsequently lyse the target cell (**Figure 1.8**). Additionally, MHC class I molecules regulate natural killer (NK) cell activity through immune receptors (**Figure 1.9**) and may bind similar receptors on other immune cell types. For example, reduction in MHC class I levels due to pathogen-mediated interference may decrease signalling through inhibitory NK cell receptors and therefore lower the threshold for NK cell activation.

### 1.2 Classical and non-classical HLA class I

HLA class I molecules are subdivided into two groups: classical HLA class Ia (HLA-A, B and C) and non-classical HLA class Ib (HLA-E, F and G). Classical HLA class I molecules are expressed on virtually every nucleated cell and are encoded by the most polymorphic genes in humans, with 5092 HLA-A, 6311 HLA-B, and 4858 HLA-C molecules described to date (IPD-IMGT/HLA Database, numbers from the latest version – 3.61 (2025–07)). Most polymorphisms are located near the peptide binding groove, which determines peptide specificity, allowing different HLA class I molecules to display different peptides for immunosurveillance. The polymorphic nature of the HLA class I genes enables selective advantage against the diversity of pathogens and antigens encountered by the population as a whole and have substantial impact on an individuals’ susceptibility to infectious diseases, cancer, and autoimmune conditions. For example, specific HLA class I alleles have been associated with susceptibility or protection from human immunodeficiency virus (HIV) disease progression (34, 35). Furthermore, several HLA-B*27 allotypes are associated with high susceptibility to ankylosing spondylitis (36–38).

Group HLA class Ib molecules display reduced genetic diversity in the human population compared to HLA class Ia molecules (IPD-IMGT/HLA Database). HLA-E is widely expressed (39), whereas HLA-F molecules are mostly expressed in lymphocytes (40–43) and HLA-G expression is restricted to human extravillous trophoblasts (EVT) (44, 45). HLA class Ib molecules are best known as NK cell ligands (46–52), however, the role and importance of non-classical HLA class I molecules on the outcome of viral infections remains largely unknown.

### 1.3 The HLA class I pathway in virus infections

While CD8^+^ T cells don’t provide sterile immunity, they are pivotal in viral infections by clearing infected cells and may impact disease duration, severity and spread. NK cells similarly play an important role in virus infections through cytotoxic activity and cytokine production (53). General disruption of host cell function during viral infections may affect HLA class I antigen processing and presentation (APP) and consequently antiviral immune responses. For example, viruses may cause host shut-off; halting synthesis of cellular proteins, including APP proteins, to favour production of viral proteins (54). Furthermore, viruses such as influenza A virus (IAV), poliovirus, coronaviruses, hepatitis C virus (HCV), and HIV-1 encode viroporins that integrate into the host (ER) membranes, thereby disrupting the ion homeostasis and inhibiting host trafficking through the secretory pathway (55). These disruptions of the infected cell may impact the abundance of peptide: HLA class I complexes at the cell surface and the quality of the peptide repertoire presented to immune cells. Many viruses have evolved targeted mechanisms to modulate HLA class I or components of the HLA class I APP pathway to evade cytotoxic immune responses. Here, we review the literature on targeted manipulation of proteins in the HLA class I APP pathway by viruses infecting humans.

## 2 Viral interference with classical HLA class I

HLA-A, -B, and -C are essential for the activation of anti-viral CD8^+^ T cells and regulate NK cell activity. While about a third of the HLA-A and -B allotypes can be recognised by killer cell immunoglobulin-like receptors (KIR), all HLA-C molecules serve as ligand for these NK cell receptors (56). These functional differences may explain selective virus-mediated downregulation of HLA-A and -B to mediate CD8^+^ T cell evasion whereas HLA-C expression remains unchanged to maintain an inhibitory signal to NK cells. Selective targeting of HLA-A and -B molecules has been reported for human cytomegalovirus (HCMV) proteins US2 & US11 (57–59), Kaposi’s sarcoma-associated herpesvirus (KSHV) protein K5 (60), human papillomavirus strain 16 (HPV-16) protein E5 (61), Epstein-Barr virus (EBV) protein BILF1 (62), adenovirus protein E3-19K (63, 64) and HIV-1 protein Nef (targeting HLA-A more than HLA-B) (65, 66). However, more recent work using primary HIV-1 strains instead of lab-adapted strains indicated that the Vpu protein of some strains can downregulate HLA-C (67, 68). Furthermore, HCMV proteins US3 and US6 expressed by recombinant vaccinia virus (rVACV) downregulate HLA-C on trophoblasts (69) and US10 was recently shown to downregulate tapasin-dependent HLA-B molecules and also HLA-C (70). KSHV also expresses a second HLA class I-modulating protein (K3) to downregulate HLA-C (60) resulting in reduced surface expression of all classical HLA class I molecules.

In contrast, VACV infection resulted in downregulation of HLA-C, while HLA-A and -B expression levels remained largely unchanged (71, 72). H3N2 influenza A virus (IAV) also seemed to cause stronger downregulation of HLA-C allotypes compared to HLA-A and -B, whereas influenza B virus (IBV) downregulated all three allotypes similarly (73). Finally, HLA-C molecules were also highly susceptible to modulation by herpes simplex virus-2 (HSV-2) protein ICP47, whereas HLA-B surface expression largely remained unaffected and varying results were shown for HLA-A (74, 75). It was proposed that HSV-2 utilises the NK cell response activated through downregulation of HLA-C to mediate killing of dendric cells and prevent activation of adaptive immunity (74).

Taken together, virus-mediated modulation of HLA class I expression is highly context dependent. Differential – e.g. time or cell type-dependent – expression of these viral proteins may regulate HLA class I modulation and therefore contribute to protection from immune recognition.

While HLA class I molecules are a common target for a broad range of viruses, there are differential mechanisms underlying viral manipulation (**Table 1**):

**Table 1:** Strategies for viral modulation of classical HLA class I.

Virus	Viral protein	Mechanism	Section	Literature
Adenovirus	E3-19K	ER retrieval of HLA class I via a dilysine motif in the cytoplasmic tail and static retention mediated by the transmembrane domain	2.3	(107–112)
Adenovirus type 12	E1A	Inhibits HLA class I transcription	2.1	(82–85)
CPXV	CPXV203	Retains HLA class I in the ER by retrieval through KTEL motif and cellular KDEL pathway	2.3	(116, 117)
CVB3	3A	Disrupts the Golgi to inhibit anterograde transport of HLA class I	2.3	(120, 121)
CVB3	2B and 2BC	Enhances HLA class I endocytosis from cell surface	2.4	(120, 121)
EBOV	GP	Glycan-mediated shielding of HLA class I at the cell surface	2.6	(161–163)
HHV-3 (VZV)	ORF66	HLA class I retention in the Golgi	2.3	(122–124)
HHV-4 (EBV)	BILF1	HLA class I retention in the Golgi	2.3	(125)
HHV-4 (EBV)	BILF1	Enhances HLA class I endocytosis from cell surface followed by lysosomal degradation	2.4	(62, 125, 131)
HHV-4 (EBV)	gp150	Glycan-mediated shielding of HLA class I at the cell surface	2.6	(164)
HHV-5 (HCMV)	US2	Binds the HC in the ER and dislocates the complex to the cytosol for proteasome-dependent degradation	2.2	(58, 95)
HHV-5 (HCMV)	US11	Binds the HC in the ER and dislocates the complex to the cytosol for proteasome-dependent degradation	2.2	(93, 94)
HHV-5 (HCMV)	US10	Prevents tapasin-dependent HLA-B from associating with PLC and stabilises and retains HLA-C in ER	2.3	(70, 106)
HHV-5 (HCMV)	US3	Retains HLA class I in ER via tapasin binding	2.3	(99–103)
HHV-6 & -7	U21	Redirects HLA class I to the lysosome	2.5	(156–160)
HHV-8 (KSHV)	vIRF1 (lytic)	Inhibits HLA class I HC transcription by blocking cellular IRF1 and possibly NFκB	2.1	(90)
HHV-8 (KSHV)	vFLIP (latent)	Enhances HLA class I transcription by stimulating NFκB	2.1	(90)
HHV-8 (KSHV)	K3	Ubiquitinates HLA class I, causing internalisation and lysosomal degradation	2.4	(60, 132, 134)
HHV-8 (KSHV)	K5	Ubiquitinates HLA class I, causing internalisation and lysosomal degradation	2.4	(60, 132, 133)
HIV-1	Vpu	Inhibits HC transcription by stabilisation of IkB, which prevents NFkB from translocating to the nucleus	2.1	(78, 79)
HIV-1	Nef	Enhances HLA class I endocytosis to TGN and sorting to lysosome. Potentially also diverts newly synthesised HLA class I from TGN.	2.4	(135, 141, 151, 154)
HPV	E7	Inhibits HLA class I transcription	2.1	(87, 88)
HPV	E5	Retains HLA class I in the Golgi	2.3	(61, 126, 127)
IAV H5N1	unknown	May inhibit HC transcription through a combination of epigenetic modulations	2.1	(92)
Seasonal IAV	unknown	Global loss in HLA class I expression which depends on the proteasome	2.5	(73)
IBV	unknown	HLA class I retention in undefined vesicular location and proteasomal degradation	2.3	(73)
MERS-CoV	unknown	May inhibit HC transcription due to alterations DNA methylation	2.1	(92)
Poliovirus	3A	Inhibits secretory pathway	2.3	(119)
SARS-CoV-2	ORF6	Inhibits HC transcription by blocking nuclear localisation STAT1, IRF1 and NLRC5	2.1	(91)
SARS-CoV-2	ORF7a	Competes with β2m for HC binding and retains molecules in the ER	2.2	(97, 98)
SARS-CoV-2	ORF8	Induces autophagy to direct HLA class I from ER to lysosome	2.5	(155)

### 2.1 Interference with HLA class I heavy chain synthesis

**Figure 2:**
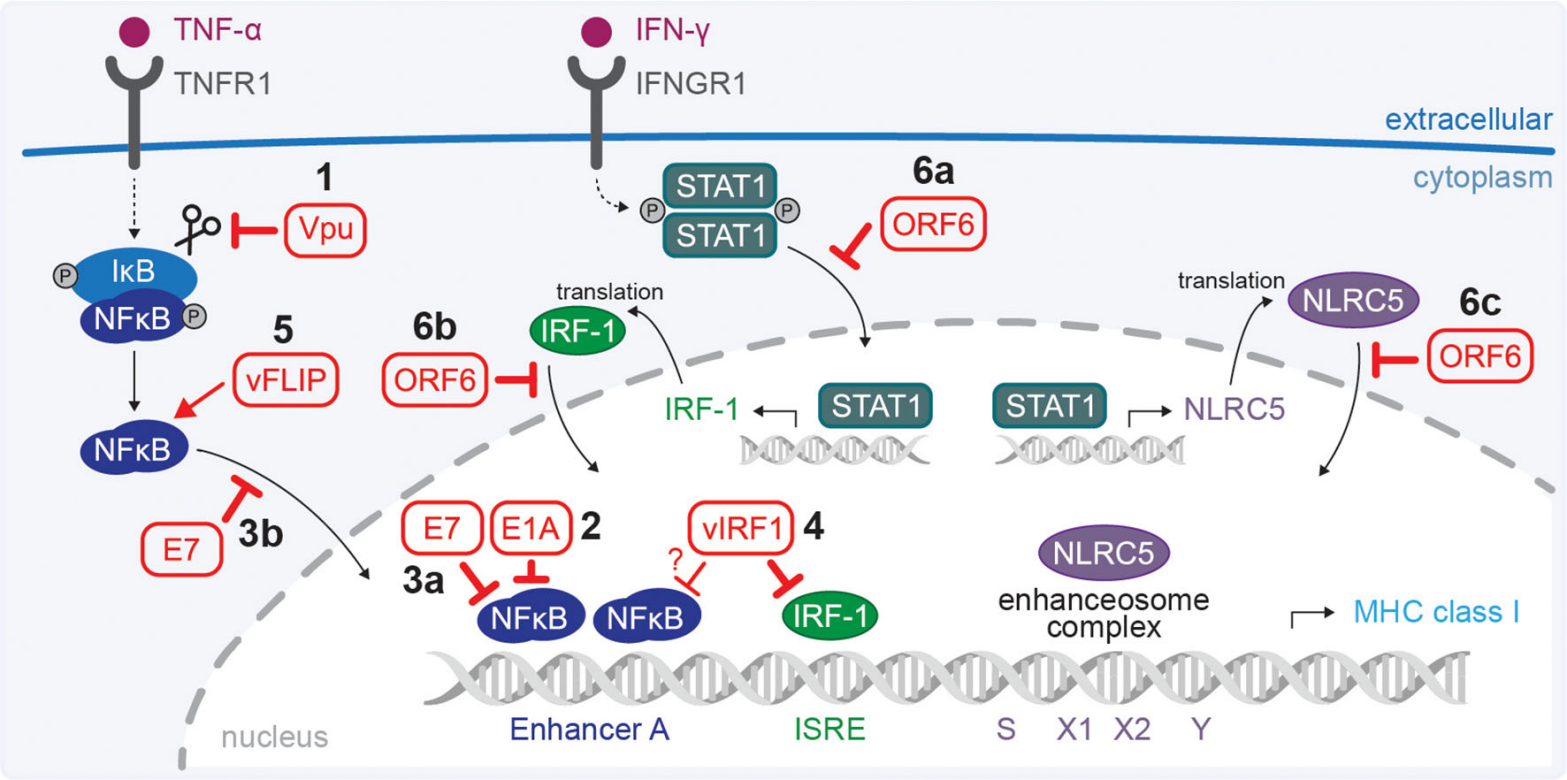
Viral interference with HLA class I HC transcription. Immune receptor signalling triggers production of transcription factors which translocate to the nucleus. NFκB, interferon regulatory factor (IRF)-1, and NOD-like receptor family CARD domain containing (NLRC) 5 regulate transcription of HLA class I genes. **(1)** IκB degradation is required to release NFκB, allowing it to translocate to the nucleus. HIV-1 Vpu stabilises IκB by inhibiting its degradation. **(2)** Ad12 E1A may disable NFκB binding and enable repressor activity. **(3)** HPV E7 may interfere with NFκB translocation (a) and repress chromatin activation (b). **(4–5)** KSHV inhibits HLA class I transcription by blocking IRF-1, and possibly NFκB, using viral IRF1 (vIRF1) **(4)**. During the latent phase KSHV expresses vFLIP to stimulate NFκB and enhance HLA class I expression **(5)**. **(6)** SARS-CoV-2 ORF6 inhibits translocation of STAT1 (a), IRF1 (b) and NLRC5 (c) into the nucleus. ISRE = interferon responsive element. DNA strand illustration from NIAID NIH BIOART Source (bioart.niaid.nih.gov/bioart/123).

Transcription of the HLA class I heavy chain can be upregulated by immune receptor signalling, for example by binding of cytokines interferon (IFN)-γ and tumour necrosis factor (TNF)-α to their respective receptors at the cell surface. The subsequent intracellular signalling leads to degradation of IκB, releasing NFκB to translocate to the nucleus where it binds to the HLA class I HC promotor region (76, 77) (**Figure 2**). Additionally, phosphorylation and homodimerisation of STAT1 in the cytoplasm is triggered. The STAT1 homodimer translocates to the nucleus where it induces expression of interferon regulatory factor (IRF)-1 and NOD-like receptor family CARD domain containing (NLRC) 5. In turn, these function as transcription factors for the expression of proteins of the HLA class I APP pathway, including the HLA class I HC (76, 77) (**Figure 2**).

Several viruses modulate different steps that regulate HLA class I transcription. For example, the HIV-1 protein Vpu, which is expressed at a late stage of infection, prevents the degradation of IκB and inhibits NFκB from translocating to the nucleus (78) (**Figure 2.1**). This results in a reduction of NFκB-dependent expression of IFN and IFN-stimulated genes, including HLA class I (78, 79). The HIV-1 Nef protein boosts NFκB expression early during the viral life cycle when Vpu is not yet expressed (78). This may explain why HIV-1 Nef presents an additional strategy to downregulate HLA class I molecules (more in section 2.4).

Oncogenic adenovirus type 12 (Ad12) encodes protein E1A that reduces MHC class I levels (80, 81). An initial report described that E1A in interferes with p105-NFκB1 processing, preventing the formation of NFkB dimers (82). Later publications describe NFκB activity in the nucleus, and that the N-terminus of Ad12 E1A prevents phosphorylation of the p65 subunit of NFκB to inhibit binding to the enhancer element (83, 84) (**Figure 2.2**). Additionally, E1A was suggested to associate with the enhancer region to recruit histone deacetylases (HDAC) 1 and HDAC8 to deacetylate histones and repress MHC class I transcription (85). The decrease in MHC class I expression due to one or more of these mechanisms may contribute to immune escape by Ad12-transformed cells.

Similar to Ad12 E1A, the oncoprotein E7 of selected HPV types was suggested to associate with the HLA class I promotor together with HDACs to repress chromatin activation (86–88) (**Figure 2.3a**). Additionally, it was reported that E7 impairs nuclear translocation of NFκB to downregulate HLA class I promotor activity (88) (**Figure 2.3b**). The E7-mediated interference with HLA class I expression may impact the susceptibility to NK cells (89).

Manipulation of HLA class I transcription is carefully balanced by oncovirus KSHV, also known as human herpesvirus 8 (HHV-8). After primary infection, herpesviruses enter a latent phase during which viral protein expression is minimal to evade immune recognition allowing the virus to persist for the lifetime of the host. The virus can reactivate from this latent phase and enter a lytic phase during which novel viral particles are produced, and cell lysis is triggered to facilitate virion release. The KSHV protein viral (v)IRF1 inhibits HLA class I expression by blocking the cellular IRF1 and possibly NFκB (90) (**Figure 2.4**). In contrast, viral FLICE inhibitory protein (vFLIP) enhances HLA class I transcription by stimulating NFκB (**Figure 5.5**), which may present a strategy to prevent uncontrolled viral dissemination in latency (90).

Severe Acute Respiratory Syndrome Coronavirus 2 (SARS-CoV-2) protein ORF6 targets the nuclear localisation of STAT1, IRF1 and NLRC5 by blocking karyopherin-mediated protein import (**Figure 2.6**). This results in reduced transcription and consequently lower HLA class I expression in SARS-CoV-2-infected cells (91). The related virus Middle East Respiratory Syndrome Coronavirus (MERS-CoV) downregulates RNA levels of genes involved in antigen presentation, both within the MHC locus and antigen presentation genes located on other chromosomes, resulting in downregulation of HLA class I proteins (92). It was proposed that this is caused by alterations in DNA methylation after MERS-CoV infection (92).

Similarly, avian H5N1 influenza virus (A/influenza/Vietnam/1203/2004) downregulated RNA expression of genes involved in antigen presentation, while seasonal H1N1 did not. In contrast to MERS-CoV infection, this led to downregulation of HLA-A and -C, but not HLA-B proteins (92). A combination of DNA methylation and histone alterations were suggested to underly the altered expression levels (92). Of note, these studies are based on -omics datasets and require further investigations to biochemically and mechanistically study altered protein expression.

### 2.2 Targeting of nascent HLA class I chains

After transcription, the mRNA encoding the HLA class I HC is translated into the ER. HCMV (or HHV-5, typically asymptomatic except in immunocompromised individuals or newborns) employs a range of strategies to evade CD8^+^ T cells and targets HLA class I HCs with US2 and US11 (58, 93–95). The US2:HC or US11:HC complexes are dislocated to the cytoplasm through the cellular Sec61 translocon where they are degraded by the proteasome (94–96) (**Figure 3.1**). A different strategy is employed by SARS-CoV-2, which encodes ORF7a to act as a β2m mimic to compete for HC binding (**Figure 3.2**). This prevents PLC formation and retains HLA class I molecules in the ER (97, 98).

**Figure 3:**
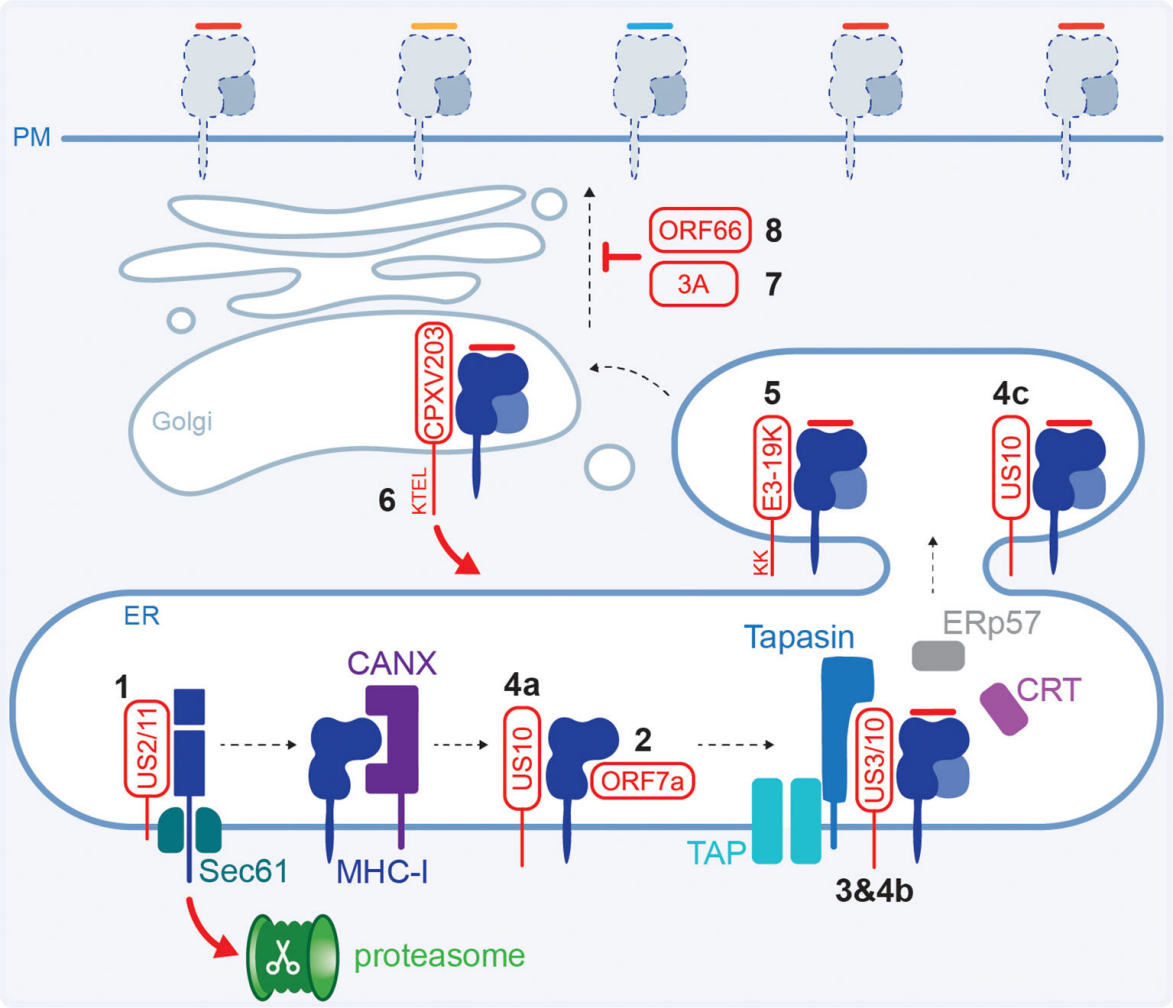
Viral interference with the HLA class I HC and transport. **(1)** HCMV US2 or US11 form a complex with the HC and target it for proteasomal degradation in the cytoplasm. **(2)** SARS-CoV-2 ORF7a mimics β2m and competes for HC binding. **(3)** HCMV US3 retains HLA class I in the ER trough tapasin binding. **(4)** HCMV US10 binds all HLA class I HCs (a) but only prevents β2m:HLA-B from interacting with the PLC (b) and interacts with selected β2m:HLA-C causing ER retention (c). **(5)** Adenovirus E3-19K retains HLA class I in the ER using a dilysine motif. **(6)** Cowpoxvirus CPXV203 has a C-terminal KTEL motif that is recognised by the cellular KDEL pathway for protein retrieval from the Golgi back to the ER. **(7)** Poliovirus and coxsackievirus B3 proteins 3A disrupt the Golgi and interfere with HLA class I trafficking. **(8)** VZV ORF66 retains HLA class I molecules in the Golgi.

### 2.3 Intracellular retention of HLA class I

Retention of HLA class I inside the cell reduces the available peptide:HLA class I complexes at the cell surface that are monitored by immune cells. Different stages of the egress pathway are targeted by viruses to downregulate surface HLA class I levels. For example, HCMV protein US3 causes ER retention of stable HLA class I heterodimers loaded with a peptide (99, 100). Distinct domains in US3 are involved in the interaction with HLA class I and binding to tapasin causing ER retention (101–103) (**Figure 3.3**). US3 is only expressed during the early phases of infection and therefore targets the existing mature pHLA class I complexes, while expression of US2 and US11 (section 2.2) peaks slightly later ensuring efficient downregulation of newly synthesised HCs. Co-expression of US3 and US2, but not US11, enhanced the association between US2 and HCs, thereby increasing their degradation (104, 105).

Additionally, HCMV protein US10 was shown to bind a diverse range of HLA class I allotypes and decreased surface expression of tapasin-dependent HLA-B, and selected HLA-C molecules (70, 106). In the proposed model, US10 interacts with all HLA class I HCs, but only tapasin-dependent HLA-B molecules are prevented from interacting with PLC components, while HLA-C:β2m heterodimers are stabilised and retained in the ER (70) (**Figures 3.4a–c**).

ER retention is also mediated by adenovirus protein E3-19K, which binds the luminal domain of MHC class I molecules (107–110). Inhibition of transport is mediated by a dilycine motif in the E3-19K cytoplasmic tail which, in concert with the transmembrane domain, enables ER retrieval for static ER retention (109, 111, 112) (**Figure 3.5**). This results in a decrease in surface HLA class I molecules and reduced CD8^+^ T cell recognition of infected cells (107, 108, 113, 114).

Using mouse cells, it was shown that cowpox virus (zoonotic infection that causes large blisters in the skin) protein CPXV203 interacts with MHC class I and has a C-terminal KTEL motif that resembles the KDEL motif in calreticulin (**Figure 3.6**). This enables CPXV203 to hijack the cellular pathway used for ER retention where calreticulin binds suboptimally loaded MHC class I in the Golgi and recruits the KDEL receptor to initiate retrograde transport (115). CPXV203-mediated downregulation of surface MHC class I levels impaired the antiviral CD8^+^ T cell response (116–118).

Even though picornaviruses are small RNA viruses, and have limited genomic capacity for immune modulatory proteins, they manipulate HLA class I molecules. While the surface expression of MHC class I was not affected, poliovirus (the causative agent of paralytic polio disease) protein 3A inhibits the secretory pathway and slows down transport of newly synthesised MHC class I molecules (119). Furthermore, coxsackievirus B3 (CVB3, does not cause serious disease in immunocompetent individuals but is more dangerous for newborns and can cause myocarditis) protein 3A disrupts the Golgi to inhibit anterograde transport (120) (**Figure 3.7**). This resulted in an impaired antiviral CD8^+^ T cell response in chimpanzee cells or mice, respectively (119, 121).

Herpesviruses other than HCMV manipulate the levels of surface HLA class I as well, although the exact molecular mechanisms have not been established. Varicella zoster virus (VZV, or HHV-3), the causative agent of chickenpox and shingles, encodes the ORF66 protein kinase that downregulates surface HLA class I by delaying maturation and mediates Golgi retention (122–124) (**Figure 3.8**). Furthermore, interference with the exocytic pathway and Golgi retention is reportedly mediated by EBV (or HHV-4, the most common cause of mononucleosis) protein BILF1, which impairs CD8^+^ T cell activation (125).

Downregulation of HLA class I from the cell surface by interference with the exocytic pathway and Golgi retention has also been reported to be mediated by protein E5 of selected HPV types (61, 126, 127). This impacted CD8^+^ T cell activation (128). Finally, it was proposed that during IBV infection, HLA class I molecules are retained in an undefined vesicular location enroute to the plasma membrane resulting in their proteasome-dependent downregulation (73). The molecular mechanism, biological consequence or *in vivo* relevance of the observed HLA class I modulation by HPV and IBV have not established thus far.

### 2.4 Internalisation of HLA class I

While CV3B protein 3A was shown to inhibit anterograde transport (section 2.3), this did not result in a complete block of HLA class I trafficking to the cell surface (120). To aid evasion of the CD8^+^ T cell response, the virus encodes proteins 2B and 2BC to enhance endocytosis resulting in removal of HLA class I from the cell surface (120, 121) (**Figure 4.1**). The mechanism underlying this surface downregulation and the fate of HLA class I after internalisation by CXV3B remain to be determined. As a result of HLA class I downregulation, very few virus-derived peptides are presented, and they display limited recognition by CD8^+^ T cells in CVB-positive individuals (129).

**Figure 4:**
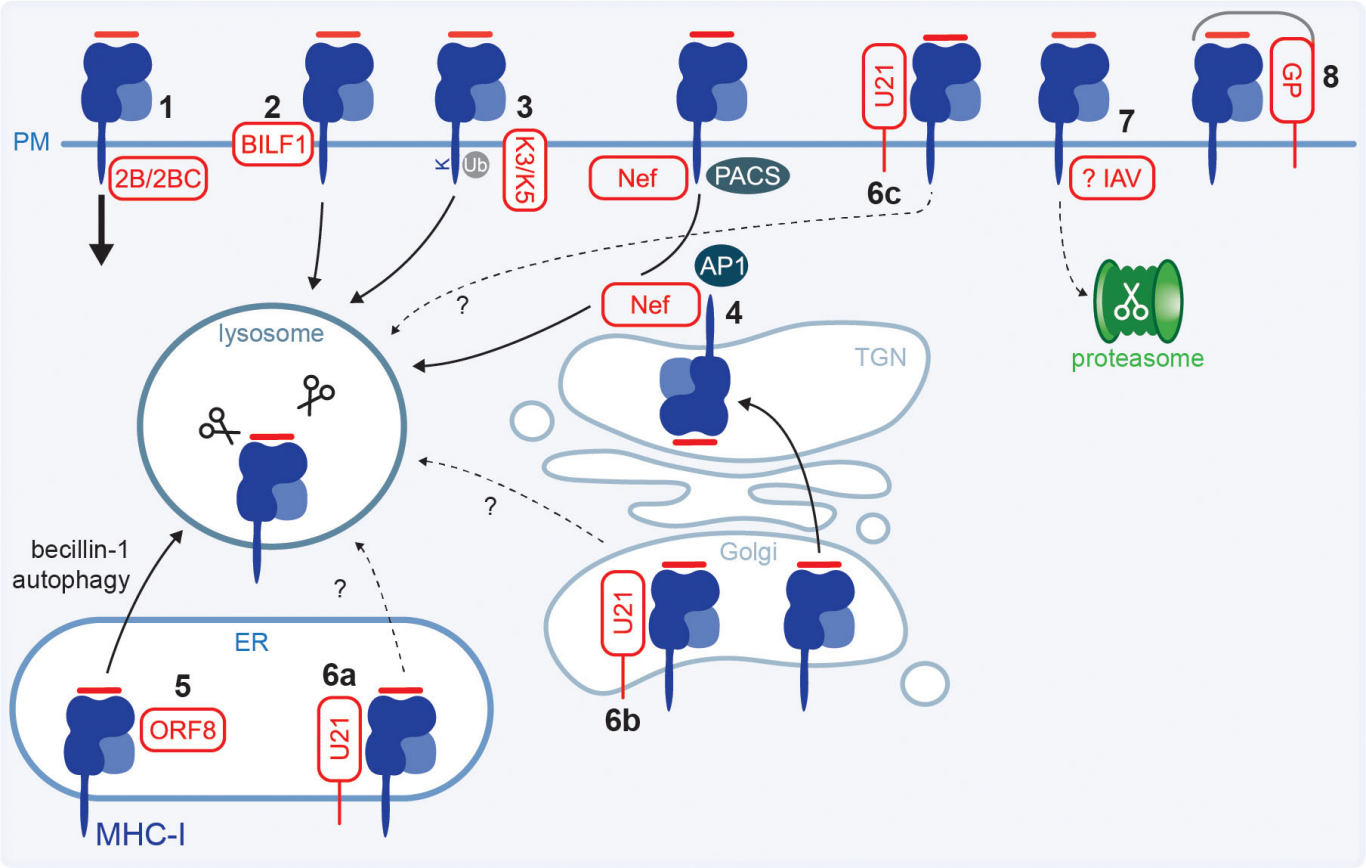
Viral proteins causing HLA class I internalisation, degradation or shielding. **(1)** CXV3B proteins 2B and 2BC enhance endocytosis of surface HLA class (I) **(2)** EBV protein BILF1 internalises surface HLA class I and directs it to the lysosome for degradation. **(3)** KSHV K3 and K5 ubiquitinate the HLA class I cytoplasmic tail which induces internalisation and trafficking to the lysosome. **(4)** HIV-1 Nef enhances HLA class I endocytosis and translocates it to the trans-Golgi network (TGN) where it accumulates and may be transported to the lysosome for degradation. It has also been proposed that Nef targets exocytic export of newly synthesised HLA class I to the lysosome. **(5)** SARS-CoV-2 ORF8 causes autophagy via the becillin-1 pathway, resulting in lysosomal HLA class I degradation. **(6)** HHV-6 and -7 encode protein U21 which redirects HLA class I to the lysosome. However, it is unknown if this occurs from the ER (a), Golgi (b) or cell surface (c). **(7)** IAV infection results in a global loss of HLA class I molecules which is dependent on the proteasome. The viral protein(s) involved has yet to be identified. **(8)** The heavily glycosylated EBOV GP and EBV gp150 shield surface HLA class I.

The EBV G protein-coupled receptor (GPCR) BILF1 similarly targets both endocytic (section 2.3) and exocytic pathways. Residues in the extracellular bridges between transmembrane domains of BILF1 were shown important for interaction with surface HLA class I, likely due to their role in maintaining BILF1 protein conformation (130). HLA class I downregulation by BILF1 is independent of its GPCR signalling properties (130, 131). BILF1 enhances HLA class I endocytosis via the DRY-like EKT motif, which is a highly conserved sequence in GPCRs located on the cytoplasmic side of transmembrane domain 3 (125). Subsequent lysosomal degradation was shown to depend on the BILF1 C-terminus and several residues in the HLA class I cytoplasmic tail (62, 125, 131) (**Figure 4.2**), although the exact motif and trafficking routes have not been identified thus far.

KSHV encodes proteins K3 and K5 that enhance endocytosis of surface HLA class I (60, 132, 133). These proteins are ubiquitin ligases that ubiquitinate a lysine in the HLA class I cytoplasmic tail, which acts as a signal for internalisation after which HLA is sorted to the late endosomal pathway where it is degraded (134) (**Figure 4.3**).

HLA class I manipulation by HIV-1 protein Nef has been extensively studied. Nef binds the HLA class I cytoplasmic tail and its acidic ^62^EEEE^65^ motif recruits cellular sorting proteins phosphofurin acidic cluster sorting protein (PACS)-1 and PACS-2 to promote retrieval from the cell surface (135–141). The methionine at position 20 in Nef prevents HLA class I from recycling back to the cell surface and redirects it to the trans-Golgi network (TGN), which acts as a sorting compartment (139). In the TGN, the Nef ^72^PXXP^75^ motif activates Src family tyrosine kinase (SFK) to stimulate phosphoinositide 3-kinase (PI3K) signalling to increase the rate of endocytosis of HLA class I from the cell surface via an ADP ribosylation factor 6 (ARF6)-regulated pathway (139, 140, 142). However, there are also reports that Nef-induced endocytosis of HLA class I is independent of PACS-1 or ARF6 and that PI3K-signalling is only required for Golgi retention rather than endocytosis (143, 144).

Binding of Nef to the HLA class I cytoplasmic tail compensates for an incomplete sorting motif in HLA class I, forming a docking site for the cellular clatherin adaptor protein (AP)-1 to sort proteins to lysosomal compartments (136, 138, 145–149). However, there are conflicting reports suggesting that the Nef:MHC class I complex is retained in the TGN without lysosomal degradation (139, 141) or may be held in a pre-lysosomal compartment but not the TGN (144). Additionally, it has been proposed that newly synthesised HLA class I, rather than molecules endocytosed from the cell surface, are trafficked from the TGN to the lysosome (146, 150). One model does not necessarily exclude the other, and Nef may indeed both internalise HLA class I and interfere with anterograde transport (151). The inconsistent reports on the source and fate of these HLA class I molecules may potentially be explained by the cell type and/or intracellular Nef concentrations used in the various studies (152, 153). Nonetheless, the reduction in surface HLA class I protects the infected cells from CD8^+^ T cell recognition (154) (**Figure 4.4**).

### 2.5 HLA class I degradation

Rather than internalising surface molecules, the SARS-CoV-2 protein ORF8 triggers lysosomal degradation of HLA class I through the induction of the Becilin-1 autophagy pathway (155) (**Figure 4.5**).

The U21 protein encoded by HHV-6 and -7 (causative agents of roseola, which usually mild and self-limiting in children) also directly binds HLA class I molecules and targets them to the lysosome, which is independent of the HLA class I cytoplasmic tail (156–159). U21 traffics to the lysosome even when HLA molecules are not associated and trafficking was proposed to involve a Golgi-derived vesicle that is clatherin-independent (158, 160). Trafficking may involve a currently unknown cellular protein (158). It remains to be established if U21 targets HLA class I molecules in the ER, Golgi or at the cell surface (158) (**Figures 4.6a–c**).

Infection with seasonal IAV resulted in a global loss of HLA class I expression and a 30-40% reduction at the cell surface (73). The mild reduction overall may potentially be due to the strong HLA-C downregulation, and more subtle downregulation of HLA-A and -B. HLA class I downregulation by IAV was shown to be dependent on the proteasome, however, the exact mechanism remains to be established (73) (**Figure 4.7**).

### 2.6 HLA class I masking

The Ebola virus (EBOV) glycoprotein (GP) was shown to sterically hinder detection of HLA class I on the cell surface (161–163) (**Figure 4.8**). The glycan-mediated shielding of surface HLA class I impaired the CD8^+^ T cell response (163). Of note, these studies were not performed in the context of EBOV infection and GP was expressed using a plasmid or adenovirus vector. Similar to EBOV, the EBV-encoded gp150 protein also causes a reduction in detection of surface HLA class I, and other antigen presentation molecules, potentially by shielding the molecules through its abundantly sialylated glycans (164). Thus, HLA class I molecules still reach the cell surface; however, activation of T cell responses was prevented through a glycan shield.

## 3 Viral manipulation of non-classical HLA class I

### 3.1 HLA-E

**Figure 5:**
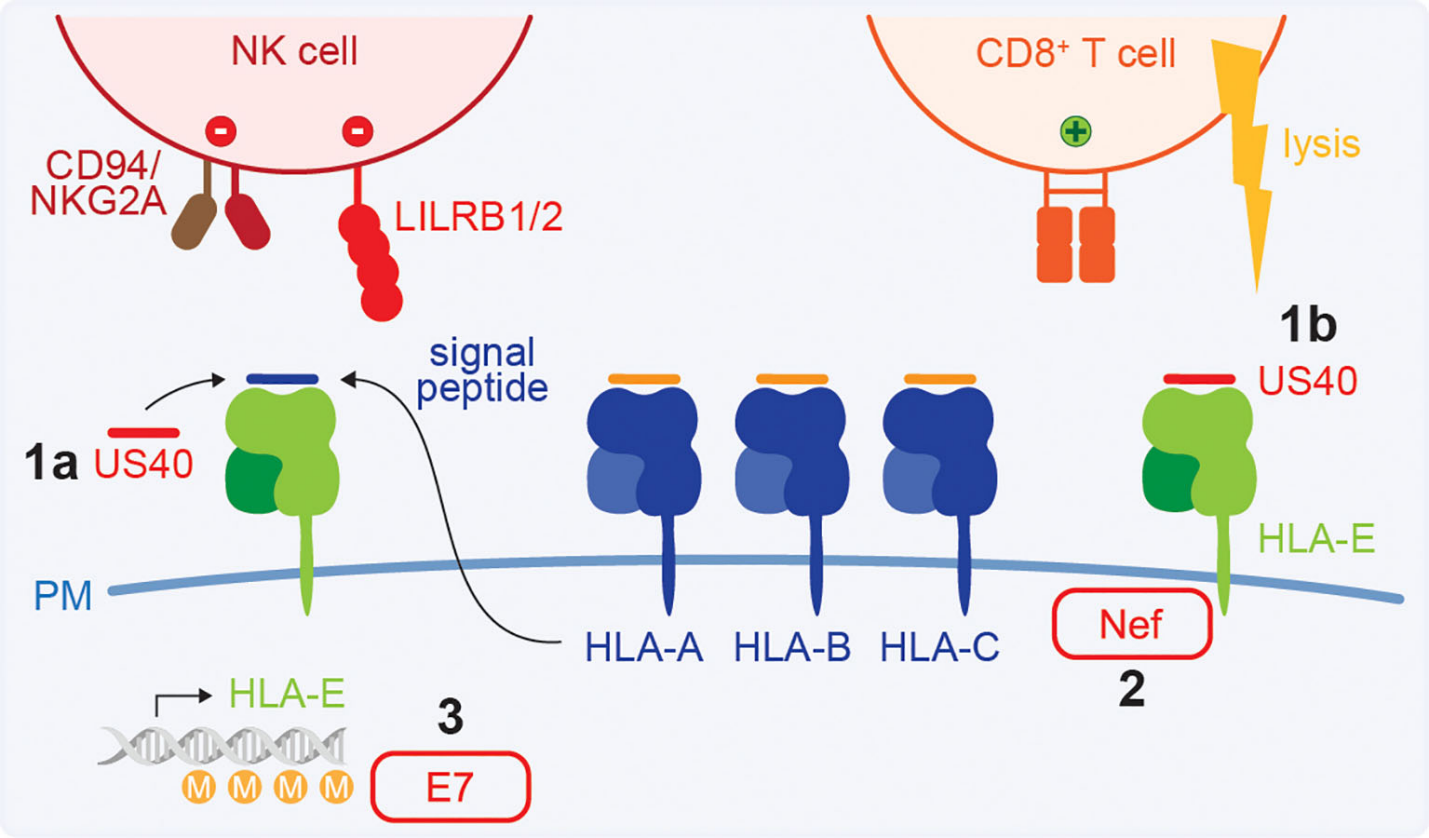
HLA-E in virus infections. HLA-E presents the signal peptide from classical HLA molecules and HLA-G, which provides an inhibitory signal to NK cells via CD94/NKG2A and LILRB1/2 receptors. HLA-E can also present peptides derived from viral proteins and activate ‘unconventional’ CD8^+^ T cell responses. **(1)** HCMV encodes a signal peptide mimic in the US40 protein, stabilising HLA-E at the cell surface and inhibiting NK cells (a). However, this can result in T cell activation (b). **(2)** HIV-1 Nef downregulates HLA-E via its cytoplasmic tail. **(3)** E7 of HPV was reported to downregulate HLA-E expression by hypermethylation of the HLA-E gene. DNA strand illustration from NIAID NIH BIOART Source (bioart.niaid.nih.gov/bioart/123).

HLA-E is widely expressed and is recognised by inhibitory CD94/NKG2 heterodimers and LILRB1/2 receptors on NK cells, and other immune cells (46–48, 165). Generally, HLA-E presents self-peptides derived from the leader sequences of HLA-A, -B, and -C molecules, which allows the immune system to monitor HLA class I expression (46, 166, 167) (**Figure 5**). This is a control mechanism for (pathogen-mediated) interference with classical HLA class I as the loss of signal peptide reduces inhibitory HLA-E levels, resulting in NK cell activation. Furthermore, HLA-E can present HLA-G-derived leader peptides. While HLA-E:peptide complexes typically have higher affinity for inhibitory CD94/NKG2A receptors, presentation of the HLA-G leader peptide results in a higher affinity for the activating CD94/NKG2C (167). Additionally, MHC-E can present virus-derived peptides, such as from HCMV, HIV, influenza virus, hepatitis B virus (HBV), HCV, and SARS-CoV-2, which activate ‘unconventional’ CD8^+^ T cell responses (168–175) (**Figure 5**). Given the low genetic variation of HLA-E in the human population, HLA-E-restricted CD8^+^ T cells are of particular interest for vaccine development (173, 176, 177).

Viral manipulation of HLA-E to evade immune cells (**Table 2**) is well-established in HCMV infection. HCMV encodes protein US40 which contains a signal peptide mimicking the signal peptide derived from cellular HLA class I molecules and is presented on HLA-E in a TAP-independent manner (178–181) (**Figure 5.1**). By stabilising HLA-E at the cell surface, HCMV compensates for the loss of HLA-A, -B, and -C expression after infection and maintains NK cell inhibitory signals (178–181). However, this can result in the activation of UL40-specific HLA-E-restricted T cell responses (168–170, 182).

**Table 2:** Viral manipulation of non-classical HLA class I.

Virus	Protein	Target	Mechanism	Section	Literature
HHV-5 (HCMV)	US40	HLA-E	Encodes a signal peptide that stabilises HLA-E at the cell surface	3.1	(178, 179) (180, 181)
HHV-5 (HCMV)	US2	HLA-G	May downregulate HLA-G, independent of the cytoplasmic tail	3.3	(57, 69, 213)
HHV-5 (HCMV)	US3	HLA-G	ER retention	3.3 (& 2.3 & 7)	(69)
HHV-5 (HCMV)	US6	HLA-G	Inhibits TAP peptide transport	3.3 (& 5)	(69)
HHV-5 (HCMV)	US10	HLA-G	Downregulates HLA-G via a tri-leucine motif in the cytoplasmic tail or ER retention	3.3	(70, 212)
HHV-6	U94	HLA-G	May act on transcription factor ATF3 to increase HLA-G expression	3.3	(209, 211)
HIV-1	unknown	HLA-F	Reduces HLA-F levels	3.2	(50, 196)
Primary HIV-1 strains	Nef	HLA-E	Downregulation by targeting the cytoplasmic tail	3.1	(183)
HPV16 & HPV18	E7	HLA-E	May reduce protein levels by hypermethylation of the HLA-E gene	3.1	(185)

In contrast, selected primary HIV-1 strains have been shown to reduce surface HLA-E expression, which was mediated by the Nef protein targeting the HLA-E cytoplasmic tail (183) (**Figure 5.2**). Modest HLA-E downregulation on B lymphoblastoid cell line (BLCL) has been reported after VACV infection, which may potentially impact NK cell activation (184). It is unclear if this is targeted downregulation mediated by VACV. Finally, HPV oncoprotein E7 of HPV16 and HPV18 strains with high oncogenic potential, but not of low-risk HPV6 and HPV11, may downregulate HLA-E expression by DNA hypermethylation of the *HLA-E* gene (185) (**Figure 5.3**). However, the effect of HLA-E downregulation on anti-HPV immune responses remains to be established.

### 3.2 HLA-F

In contrast to the highly polymorphic HLA class Ia molecules, only 122 HLA-F alleles have been described which encode 27 different HLA-F proteins (IPD-IMGT/HLA Database). The peptide binding groove of HLA-F in complex with β_2_m accommodates relatively long peptides (186–188). However, peptide loading is not essential for HLA-F trafficking and surface expression, and the protein can be expressed as an open conformer (OC) consisting of the HC only (41, 43, 189, 190). These OCs may also be expressed as a homodimer or a heterodimer with an HLA class Ia HC, potentially with a role in cross-presentation of exogenous antigens (191).

HLA-F resides mostly intracellularly but can be expressed at the cell surface of activated lymphocytes as ligands for activating and inhibitory KIR and LILR receptors on immune cells (40–43, 49, 50). For example, the activating NK cell receptor KIR3DS1 recognises HLA-F OCs while the HLA-F:β_2_m:peptide complex binds inhibitory receptor LILRB1/2 (49, 186, 192). HLA-F OCs are also recognised by inhibitory receptors KIR3DL1 and KIR3DL2, although they bind with lower affinity compared to KIR3DS1 (50, 191). Conflicting results have been published regarding HLA-F OCs functioning as a ligand for KIR2DS4 (50, 191). It is currently unknown if viral peptides can be presented by HLA-F to regulate NK or T cell responses.

Increased HLA-F expression has been reported after infection with Japanese Encephalitis virus, HIV-1 (early in infection), HCV, and BK polyomavirus (BKpV) (50, 193–195). For the latter three, this was shown to result in enhanced recognition by KIR3DS1 and increased NK cell activation *in vitro* (50, 194–196). Furthermore, KIR3DS1 has been associated with delayed progression of disease caused by HIV-1, and the HLA-F*01:03 polymorphism was associated decreased levels of HBV DNA (50, 197). Taken together, this suggests that HLA-F may play a role in infection control.

Evidence for manipulation of HLA-F by viruses to escape immune responses is limited. HLA-F expression may be reduced on CD4^+^ T cells late in infection with HIV-1, impacting KIR3DS1 binding and NK cell activation (50, 196). However, the mechanism and impact on antiviral immune responses remain elusive.

### 3.3 HLA-G

HLA-G expression is restricted to EVT cells at the maternal-foetal interface where it mediates tolerance (51, 198–200). HLA-G is the only HLA class I molecule described to form β_2_m-associated homodimers, which interact with the inhibitory LILR receptors (200–202). HLA-G has a number of additional unusual features including the lack of an endosomal recycling motif (203), presence of an ER retrieval motif (204), and it presents a restricted peptide repertoire (205, 206). Several HLA-G isoforms have been described, including soluble versions, although the biological role and relevance of the alternative transcripts *in vivo* has yet to be confirmed (207).

A recent review summarised neoexpression of HLA-G in cells infected with HPV, HBV, HCV, HCMV, EBV, HIV, human lymphotropic virus (HTLV)-1, IAV or SARS-CoV-2 (208). However, this should be interpreted with caution as i) most antibodies against HLA-G are poorly characterised and may cross-react with classical HLA molecules, ii) experimental setup may be lacking controls or an Fc receptor block, which is essential particularly when staining immune cells, and iii) the biological relevance of soluble HLA-G *in vivo* remains controversial (207). The role of HLA-G in virus infections therefore remains elusive.

Targeted manipulation of HLA-G (**Table 2**) has been reported for human herpesvirus 6 (HHV-6), which in the placenta predisposes the mother to pre-eclampsia (209, 210). HHV-6 protein U94 acts on the human transcription factor cyclic AMP-dependent transcription factor (ATF)3 and reportedly increases expression of both membrane and soluble HLA-G isoforms (211). Given the role of HLA-G in tolerance, increased HLA-G expression levels may result in suppression of the antiviral immune response. Of note, these studies did not include biochemical analyses and the 87G antibody that was used may cross-react with HLA class Ia molecules (207).

HCMV protein US10 downregulates HLA-G via a tri-leucine motif in the cytoplasmic tail in a proteasome-dependent manner (212). However, a more recent study proposed US10 binding to HLA-G to mediate ER retention without destabilisation (70). US10-mediated interference with HLA-G hampered NK cell inhibition (212). HCMV proteins US3 (sections 2.3 & 7) and US6 (more in section 5) were also reported to interfere with HLA-G expression (69). Conflicting results have been reported for US2, which have been attributed to experimental setup (57, 69, 213). All these studies include biochemical analysis detecting the smaller 39kDa HLA-G HC, opposed to the 45kDa classical HLA HC, providing confidence that the results are indeed HLA-G-specific. It has been hypothesised that HCMV-mediated downregulation of HLA-G as a ligand for inhibitory NK receptors may play a role in release of intracellular viral particles through cytolysis or that the activated NK cells may produce a more favourable cytokine environment for the virus (212).

## 4 Viral interference with peptide generation

The proteasome plays a pivotal role in the generation of antigenic peptides for presentation on MHC class I molecules through the regulated degradation of virtually all proteins in the cell (214). The proteasome core consists of four stacked heptameric rings – two α (outer) and two β (inner) rings – that together form a barrel-shape. This 20S core is present with or without regulatory subunits capping either end. The catalytic activity of the core is restricted to β1 (caspase-like), β2 (trypsin-like), and β5 (chymotrypsin-like) subunits (215). Three alternative β subunits (β1i/LMP2, β2i/MECL1, β5i/LMP7) are part of the specialised immunoproteasome that displays altered peptide-cleave properties (216). The immunoproteasome, with 11S regulatory subunit, is abundantly expressed in hematopoietic cells and can be induced in non-immune cells in inflammatory conditions such as viral infections (216).

Two herpesviruses have been described to modulate MHC class I antigen presentation by reducing the pool of available peptides in *cis*, e.g. reducing peptides derived from these two herpesvirus proteins (**Table 3**). EBV nuclear antigen 1 (EBNA1) protein expressed during latency contains a long repetitive sequence consisting exclusively of glycine and alanine residues. Initially, it was described that this prevents its degradation by the proteasome (217). However, the absence or low level of EBNA-1-derived peptides was later attributed to inhibition of messenger RNA translation in *cis* to interfere with formation of EBNA1 DRiPs (218, 219) (**Figure 6.1**). The reduced pool of EBNA1-derived peptides available for MHC class I presentation limits the EBNA1-specific CD8^+^ T cell response (220, 221). Similarly, the EBNA1 homologue latency-associated nuclear antigen 1 (LANA1) encoded by KSHV contains a QED-rich central repeat (CR) domain that inhibits translation in *cis and* impacts presentation of LANA1-derived MHC class I antigens (222, 223) (**Figure 6.2**).

**Table 3:** Viral interference with antigen processing.

Virus	Viral protein	Target	Mechanism	Literature
Adenovirus	E1A	β1i	Downregulation at transcriptional level	(230)
HBV	HBxAg	α4, 19S and 11S	Prevents the interaction of the 11S regulator with the 20S core and interacts with both α4 and PSMC1 of the 19S regulatory subunit	(232–235)
HCV	NS3	β5i	Binds subunit to reduce immunoproteasome activity	(231)
HHV-4 (EBV)	EBNA1	EBNA1	Contains long repetitive sequence of glycine and alanine residues that inhibit its processing.	(217–219)
HHV-8 (KSHV)	LANA1	LANA1	Repetitive sequence of predominantly glutamine, glutamate, and aspartate residues to inhibit processing in *cis*	(222, 223)
HIV-1	Gag p24	β2i, β5i and 11S	Downregulates subunits in dendritic cells	(224)
HIV-1	Tat	α4, α7, β(i)	Prevents the interaction of the 11S regulator with the 20S core and modifies composition of the (immuno)proteasome	(225–229)
HPV18 & 6b	E7	β1i	Represses promotor	(86)

**Figure 6:**
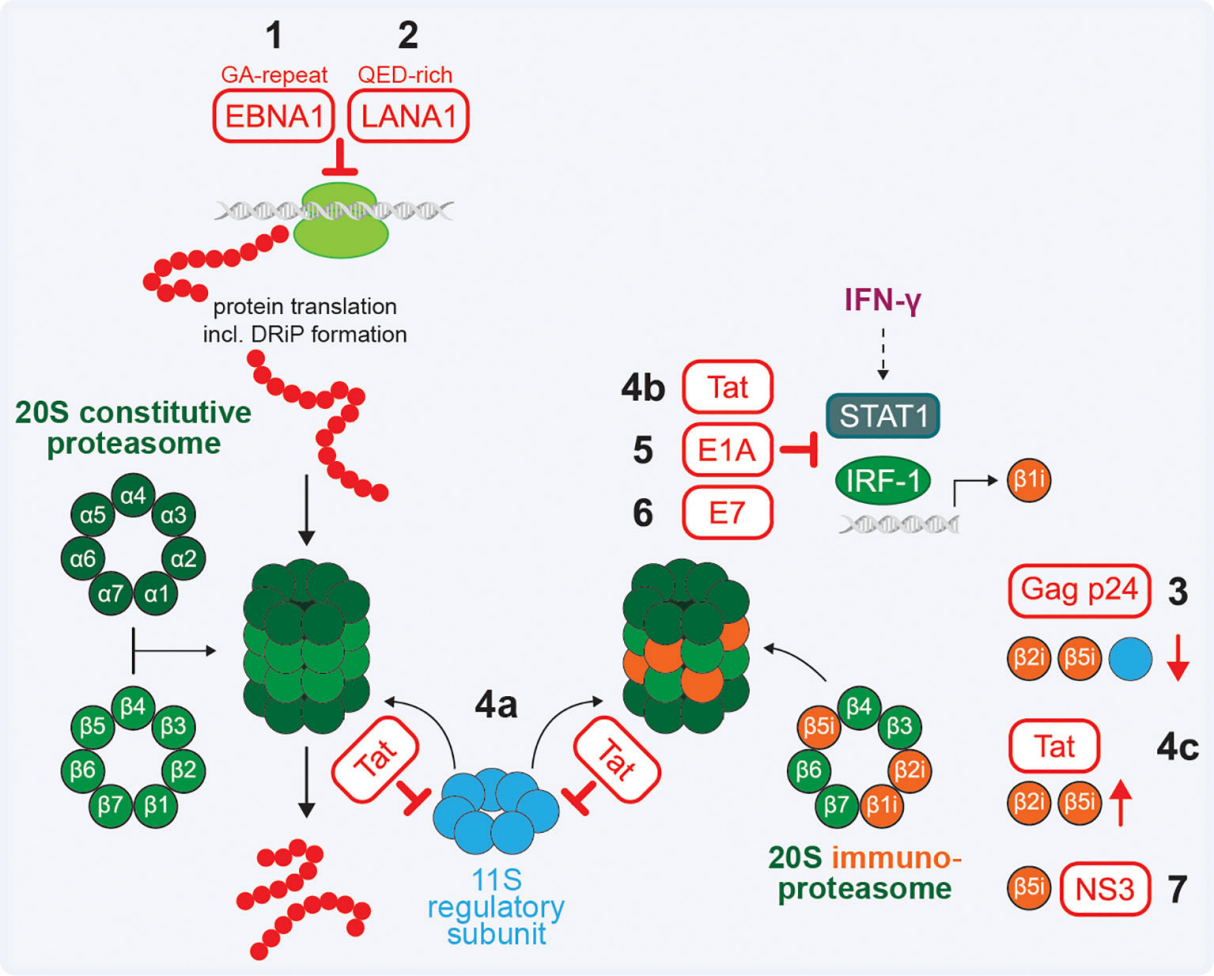
Viral interference with antigen processing. **(1–2)** EBV EBNA1 and KSHV LANA1 inhibit translation in *cis* to reduce the pool of peptides derived from these proteins. **(3)** HIV-1 Gag p24 downregulates the β2i and β5i subunits from the immunoproteasome and the 11S regulatory subunit that can associate with either the constitutive or immunoproteasome. **(4)** HIV-1 Tat prevents interaction of 11S with the 20S core (a), downregulates β1i transcription (b) and stabilises the β2i and β5i subunits (c). **(5–6)** Adenovirus E1A and HPV E7 downregulate β1i transcription. **(7)** HCV NS3 interferes with β5i to reduce immunoproteasome activity.

Virus-mediated manipulation of proteasome subunits can impact antigen processing (**Table 3**) and may result in changes of the peptide repertoire presented at the cell surface. The HIV-1 Gag p24 protein was described to downregulate the β2i and β5i subunits of the immunoproteasome and components of the 11S regulatory subunit (alias PA28) in dendritic cells (224) (**Figure 6.3**). More established is proteasome modulation by the HIV-1 Tat protein, which binds the α4 and α7 subunits to prevent the interaction of 11S with the 20S core (225–227) (**Figure 6.4a**). Additionally, Tat mediates downregulation of β1i at transcriptional level through interference with the formation of the STAT1-IRF-1 complex, inhibiting IRF-1 binding to the β1i promotor (228) (**Figure 6.4b**). Tat also interacts with six β subunits of the constitutive 20S core, and stabilises β2i and β5i, leading to a modified composition of the (immuno)proteasome (227, 229) (**Figure 6.4c**).

Similar to HIV-1 Tat, adenovirus protein E1A and protein E7 from high-risk HPV18 and low-risk strain 6b were reported to downregulate β1i expression at transcription level to interfere with the presentation of viral antigens (86, 230) (**Figure 6.5, 6**). Furthermore, HCV protein NS3 binds to β5i to reduce immunoproteasome activity (231), which may interfere with processing of viral antigens and impact antiviral immune responses (**Figure 6.7**).

HBV infection causes viral hepatitis which can lead to hepatocellular carcinoma. While HBxAg encoded by HBV is a substrate for proteasome degradation, it binds the α4 subunit of the 20S proteasome core and the PSMC1 component of the 19S regulatory subunit (232–234). As described for HIV-1 Tat, the interaction between HBxAg and α4 may inhibit binding of the 11S regulatory subunit and explain the observed proteasome inhibition (234, 235). The interactions between HBxAg and components of the proteasome have predominantly been described in the context of its role as a transactivator. The impact of HBxAg on the presented HLA class I peptide repertoire and the antiviral CD8^+^ T cell response has thus far not been reported.

## 5 Viral modulation of peptide transport by TAP

**Figure 7:**
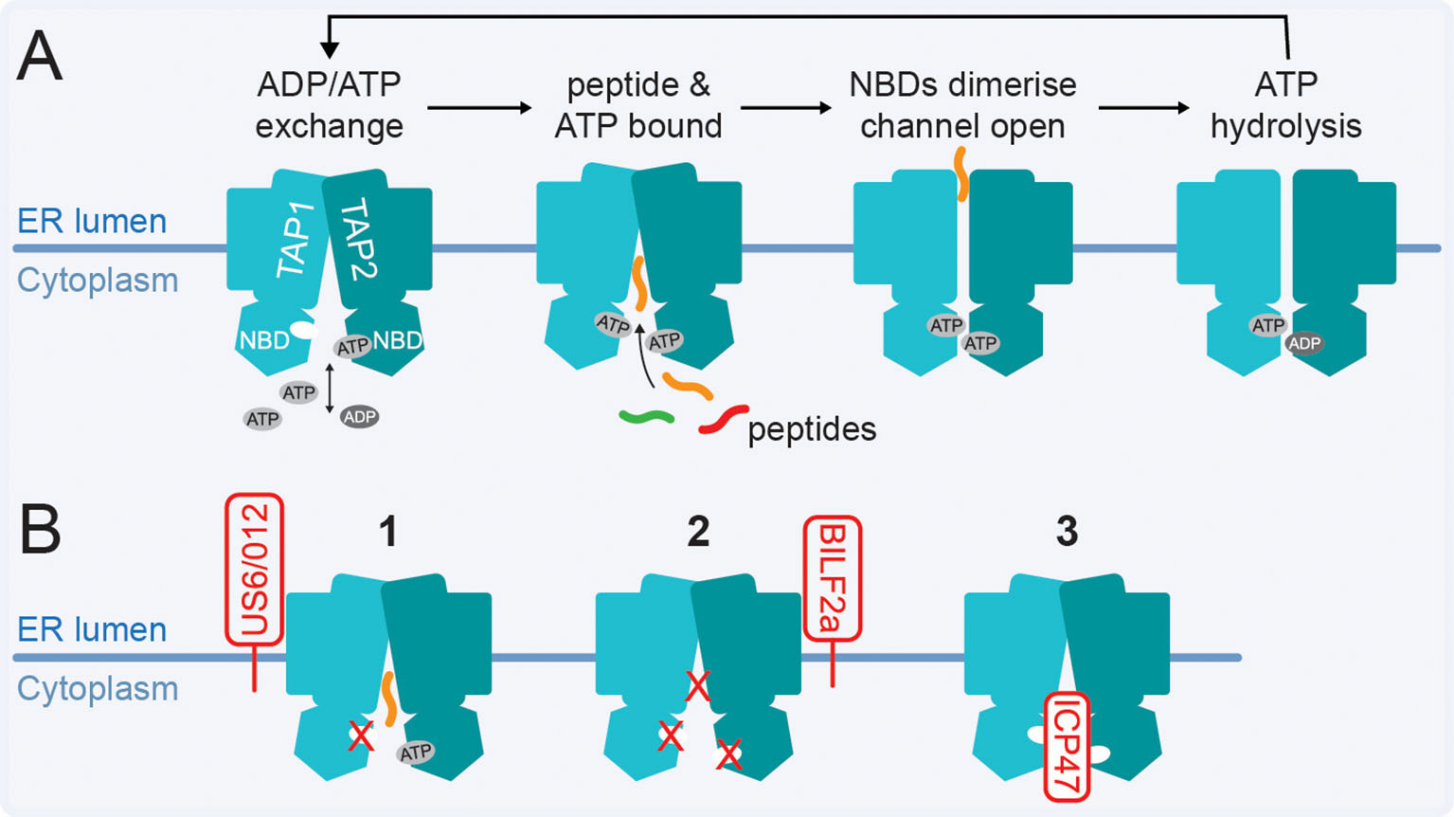
TAP-mediated peptide transport in virus infections. **(A)** Schematic overview of peptide transport by TAP. **(B)** (1) HCMV US6 and CPXV protein CPXV012 bind TAP in the ER lumen to block ATP binding, likely through distal allosteric effects. (2) EBV protein BILF2a prevents both ATP and peptide binding to the ‘inward’ facing conformation of TAP. (3) HSV-1/2 protein ICP47 binds TAP on the cytosolic side to block peptide binding by competitive inhibition.

The TAP transporter shuttles peptides from the cytoplasm into the ER and is an essential component of the PLC (**Figure 1.4**). Subunits TAP1 and TAP2 each consist of a series of transmembrane domains (TMD) with an ATP-binding C-terminal nucleotide-binding domain (NBD) in the cytoplasm (236). When the peptide binding pocket faces the cytoplasm, the NBDs are separated. Peptide and ATP bind TAP independently, and when both are bound the NBDs dimerise. These conformational rearrangements are relayed to the TMDs, exposing the peptide binding pocket to the ER lumen where the peptide is released. Upon ATP hydrolysis, the NBDs dissociate and TAP switches back to a cytoplasm (‘inward’)-facing conformation (236) (**Figure 7A**).

TAP1 is expressed using a bidirectional promotor that is shared with the β1i gene. Although the transcription factor requirement for the two genes is not identical, viral proteins manipulating β1i expression at transcriptional level (section 4) may similarly affect TAP1. For example, protein E7 of selected HPV strains was suggested to inhibit peptide transport by TAP and decrease TAP1, but not TAP2, levels potentially by transcriptional regulation (86, 237, 238).

Blocking of peptide transport from the cytoplasm into the ER is a common mechanism by which viruses, particularly herpesviruses, interfere with HLA class I antigen presentation (**Table 4**). HCMV protein US6 binds TAP in the ER lumen and prevents the conformational changes required for ATP to bind TAP1 but does not interfere with peptide binding (239–241) (**Figure 7B.1**). A similar mechanism was described for CPXV transmembrane protein CPXV012 (118, 242), resulting in reduced MHC class I surface expression and affects the antiviral CD8^+^ T cell response (117, 118).

**Table 4:** Viral manipulation of TAP.

Virus	Viral protein	Mechanism	Literature
CPXV	CPXV012	Blocks ATP binding	(118, 242)
HHV-1&2 (HSV1&2)	ICP47	Competitive inhibition to block peptide binding. Turns off ATP hydrolysis.	(245–249)
HHV-4 (EBV)	BNLF2a	Prevents ATP and peptide binding to TAP	(243, 244)
HHV-5 (HCMV)	US6	Induces conformational changes depriving TAP1 from ATP	(239–241)
HPV	E7	May inhibit TAP1 gene expression through bidirectional promotor shared with the β1i gene	(86, 237, 238)

The 60 amino acid EBV protein BILF2a also interacts with TAP in the ER lumen and interferes with ATP and peptide binding to TAP (243, 244) (**Figure 7B.2**). Finally, HSV-1 and 2 (or HHV-1&2) encode the 88 amino acid protein ICP47 that binds TAP1/2 on the cytoplasmic side and blocks peptide binding by competitive inhibition (245–248) (**Figure 7B.3**). While ATP can still bind, ICP47 freezes TAP in the ‘inward’ facing state with the NBDs unable to dimerise, which is required for ATP hydrolysis (247–249). For all these herpesviruses, reduction of the peptide supply in the ER by interference with TAP function resulted in decreased HLA class I surface expression and inhibition of recognition by CD8^+^ T cells (243, 244, 250, 251).

## 6 Viral inhibition of ERAP-mediated peptide trimming

Within the ER, ERAPs trim N-terminal extensions of peptide precursors to generate optimal length peptides for presentation on HLA class I (252). Humans encode ERAP1 and ERAP2, which share 49% sequence identity and exhibit preferential differences for their peptide substrate (14, 15, 253). Peptides of 9–16 amino acids with hydrophobic N-terminal extensions, excluding proline, have a higher affinity for trimming by ERAP1, whilst shorter peptides with basic N-termini are more efficiently trimmed by ERAP2 (253–255). The molecules can also trim peptides synergistically as a heterodimer, however, functional ERAP2 may not be expressed by 25% of individuals due to a single nucleotide polymorphism (SNP) resulting in alternative splicing and a truncated protein (15, 255, 256). Addition of recombinant ERAP2 to peripheral blood mononuclear cells (PBMCs) from healthy individuals was able to reduce HIV infection *in vitro* (257). Furthermore, ERAP1 exerts selective pressure on HIV-1 in individuals expressing HLA-B*57, which commonly results in a mutation that prevents ERAP1 from trimming an immunodominant epitope. This was associated with a 22-fold increase in viral load (258). Taken together, ERAP activity strongly impacts the (viral) repertoire presented on HLA class I molecules.

While virus-mediated manipulation of ERAP2 has thus far not been described, HCMV downregulates ERAP1 during infection using two microRNAs, miR-US4–1 and miR-UL122-5p, that target the 3’ untranslated region (UTR) of ERAP1 (259, 260) (**Table 5**). This results in recognition by the RNA-induced silencing complex, followed by degradation of the transcript (259). Decreased ERAP1 expression in HCMV-infected cells reduced trimming of virus-derived peptides and impairs T cell recognition of the infected cells (259, 260). Naturally occurring SNPs within the microRNA binding site of ERAP1 can render it resistant to downregulation by HCMV, which may be counteracted by heterogeneity within the HCMV microRNAs (260, 261).

**Table 5:** Viral manipulation of proteins impacting the peptide repertoire.

Virus	Viral component	Mechanism	Literature
Adenovirus	E3-19K	Independently binds HC and TAP to prevent PLC formation and tapasin function	(270)
HHV-5 (HCMV)	miR-US4-1 & miR-UL122-5p	microRNAs preventing ERAP1 translation	(259, 260)
HHV-5 (HCMV)	US3	Disrupts PLC formation and reduces tapasin synthesis	(103, 269, 273)
MCV	MC80	ERAD-mediated tapasin degradation	(271)

## 7 Viral modulation of peptide editing by tapasin

Tapasin is a central component of the PLC, bridging TAP with peptide-receptive HLA class I molecules to facilitate the association of high-affinity peptides (19, 25, 26, 262–264). The loss of tapasin results in an altered peptide repertoire and (antiviral) CD8^+^ T cell response (265, 266). HLA class I allotypes exhibit varying degrees of reliance on tapasin binding for their association with high-affinity peptide cargo (267). Therefore, viral modulation of tapasin (**Table 5**) may result in a reduction in surface expression of tapasin-dependent HLA class I molecules and may not affect other allotypes.

The HCMV protein US3 binds tapasin not only to retain HLA class I molecules in the cell (section 2.3) but also prevents peptide loading on tapasin-dependent HLA class I molecules (103). HCMV may regulate tapasin inhibition using a truncated isoform of US3, which functions as a dominant negative regulator of full-length US3 activity thereby abolishing the initial disruption of tapasin-mediated peptide optimisation (268). Furthermore, HCMV infection also reduces synthesis of new tapasin molecules (269).

In addition to its role in retaining HLA class I in the ER (section 2.3), adenovirus E3-19K prevents tapasin from optimising the HLA class I peptide repertoire by independently binding both HCs and TAP, preventing the formation of the PLC (270). This allows E3-19K to affect antigen presentation by HLA class I molecules that it only moderately affects by direct retention (270).

Molluscum cantagiosum virus (MCV, an endemic human poxvirus that can cause skin lesions) encodes MC80, an MHC class I-like protein that associates with β_2_m to interact with tapasin and TAP. This leads to the recruitment of ER-associated protein degradation (ERAD) complexes targeting tapasin for ubiquitination and proteasomal degradation (271). Consequently, TAP levels are also downregulated and there is a reduction in classical HLA class I and HLA-E levels (271, 272). MC80 expression from an adenovirus backbone modulated NK cell activity and promoted evasion of CD8^+^ T cells (272).

## 8 Discussion

Manipulation of HLA class I and the APP pathway by large DNA viruses such as herpesviruses has been well established over the last four decades. This work was complemented by studies on MHC evasion by non-human viruses, for example murine CMV, equine herpesvirus, and bovine herpesvirus. It has become clear that numerous different strategies to modulate MHC class I levels are used by a wide range of viruses – both DNA and RNA viruses, and viruses causing acute or chronic infections. This highlights the importance of the APP pathway in the antiviral immune response.

While for example HLA class I modulation in HCMV infection is well-established, different mechanisms have been proposed to underly other viral manipulation strategies – e.g. HLA class I modulation by the HIV Nef protein. The seemingly contradicting findings could potentially be due to the use of primary vs. laboratory-adapted strains. Alternatively, they may simply reflect temporal regulation as immune evasion requirements early in infection may differ from those at a late stage of infection. Similarly, different manipulation mechanisms may be required depending on the cell type infected or influences of the tissue environment.

Although many viruses use more than one strategy to widely modulate HLA class I expression, allotype-specific manipulation has been reported for several viral proteins. The HLA molecule-specific manipulation by viral proteins may not only be due to direct targeting of specific allotypes. It may also reflect the dependency of a given HLA molecules on components of the APP pathway, e.g. TAP or tapasin, for peptide acquisition. Therefore, viral inhibitors of the HLA class I pathway serve as useful research tools. Indeed, the discovery and characterisation of viral proteins targeting the HLA class I pathway have greatly enhanced our understanding of APP in health and disease.

Nonetheless, the role and importance of non-classical HLA class I molecules, particularly HLA-F and -G, in virus infections remains largely elusive. Historically, lack of reagents with sufficient specificity has complicated investigations into these proteins. For example, several HLA-G antibodies have been shown to cross-react with classical HLA molecules over the years (207). Furthermore, the absence of a murine HLA-F homologue has also hampered research into its role and function, including in virus infections. Excitingly, recent work on HLA-E demonstrates the tremendous potential of these molecules in vaccine development due to the non-polymorphic nature of non-classical HLA class I (173, 176, 177). Therefore, it is pivotal that we continue to enhance our understanding of the various HLA class I molecules and components of the antigen processing and presentation pathway.

Viruses such as adenoviruses, poxviruses, HSV-1 and HCMV are of interest as vector vaccine or oncolytic therapeutics. The efficiency of these viral vectors may be enhanced by strategically altering the expression of viral vector backbone proteins manipulating the APP pathway. Continued characterisation of interactions between viruses and the HLA class I APP components is therefore highly relevant for the development of novel vaccines and antivirals, with potential applications in development of cancer and autoimmunity therapeutics.
